# A comprehensive policy for reducing sugar beverages for healthy life extension

**DOI:** 10.1186/s12199-019-0767-y

**Published:** 2019-02-26

**Authors:** Yoshihiro Kokubo, Aya Higashiyama, Makoto Watanabe, Yoshihiro Miyamoto

**Affiliations:** Department of Preventive Cardiology, National Cerebral and Cardiovascular Center, 5-7-1, Fujishiro-dai, Suita, Osaka, 565-8565 Japan

**Keywords:** Sugar-sweetened beverages, Fructose corn syrup, Metabolic syndrome, Social policy

## Abstract

The excessive consumption of sugar-sweetened beverages is a public health concern worldwide. Several clinical trials examining the effects of consuming sucrose or high-fructose corn syrup demonstrated the link between this consumption and increased risk factors for cardiometabolic diseases. In this issue of *Environmental Health and Preventive Medicine*, Li et al. examined the sugar-sweetened beverage consumption among undergraduate students and evaluated the relationship between this consumption and the “late” chronotype, sleep duration, and weight increase. They concluded that the sugar-sweetened beverage intake might mediate the associations among sleep duration, late chronotype, and weight gain and that the intake of sugar-sweetened beverages in the evening may be a risk factor for the development of overweight/obesity. A systematic review and meta-analysis of prospective cohort studies and randomized controlled trials provided evidence that the consumption of sugar-sweetened beverages promotes weight gain in both children and adults. The World Health Organization guideline highly recommends reducing the intake of sugars to less than 10% of one’s total energy intake. The Dietary Approaches to Stop Hypertension diet and the Mediterranean diet were shown to help individuals refrain from sweets and sugar-containing beverages. A global evaluation revealed how much disability during accumulated lifetime hours is due to sugar-sweetened beverages. Interventions are necessary, but many individuals find it quite difficult to reduce or eliminate their high intake of sugar-sweetened beverages. The taxation of sugar-sweetened beverages was demonstrated to have a significant positive influence on individuals’ planned purchases and the probability of the purchase of healthy beverages. Western countries are working on the social regulation of sugar-sweetened beverages, but Japan has not implemented any similar regulations. The social regulation of sugar-sweetened beverages is necessary to stop the increase of diabetes morbidity and the increase in dementia that often accompanies this morbidity.

The excessive consumption of sugar-sweetened beverages is a public health concern worldwide. At the beginning of the twenty-first century, the US Department of Agriculture reported that over the past 50 years, the consumption of soft drinks per capita in the USA had risen by approx. fivefold. The typical beverages with added high-fructose corn syrup marketed in the USA are approx. 55% fructose. In Japan, the demand for isomerized sugar has been growing. According to the data maintained by Japan’s Ministry of Agriculture, Forestry and Fisheries, the sugar demands in Japan in 2005 and 2017 were 2165 kt and 1921 kt and those of isomerized sugars were 790 kt and 832 kt, respectively [1]. Approximately half of these sugar amounts are used for soft drinks, and the increase in the demand for sugar is due to soft drinks.

Several clinical trials that investigated the effects of consuming sucrose or high-fructose corn syrup demonstrated that the consumption of sugars increases an individual’s risk of developing cardiometabolic diseases [2]. The consumption of high-fructose corn syrup can have unfavorable effects including weight gain, insulin resistance, hypo-high-density lipoprotein cholesterolemia, and hypertriglyceridemia [3, 4]. Fructose from sucrose or high-fructose corn syrup enhances salt absorption in the gut and kidney and enhances intracellular angiotensin formation [5], whereas the fructose contained in fruits does not raise blood pressure because fruits’ high potassium, antioxidant vitamins such as vitamin C, various flavonols, and dietary fiber protect against this fructose-induced effect on blood pressure [6].

In this issue of *Environmental Health and Preventive Medicine*, Li and colleagues examined sugar-sweetened beverage consumption mediating the relationship between the “late” chronotype, sleep duration, and weight increase among undergraduate students [7]. Eight hundred college students (mean age 19.8 ± 1.1 years) from four universities in China participated, and Li et al. identified a significant indirect effect of sugar-sweetened beverage consumption between the student’s chronotype and body mass index (BMI) (effect = − 0.03, standard error [SE] = 0.01 [95% confidence intervals − 0.05, − 0.02]) and between the students’ sleep duration and BMI (effect = − 0.12, SE = 0.05 [− 0.16, − 0.09]). The students’ exercise level and psychological condition were also shown to have mediating effects between the chronotype and BMI (effect = − 0.04, SE = 0.01 [− 0.06, − 0.01], and effect = − 0.03, SE = 0.02 [− 0.05, − 0.01]). Li et al. concluded that (1) the sugar beverage intake might mediate the association among sleep duration, late chronotype, and weight gain in college students, and (2) an individual’s sugar beverage intake in the evening may be a risk factor for the development of overweight/obesity. Li et al. suggest that schools should take proper measures to decrease the frequency of this exposure.

A 2013 systematic review and meta-analysis of prospective cohort studies and randomized controlled trials provided evidence that the consumption of sugar-sweetened beverages promotes weight gain in both children and adults [8]. Another systematic review and meta-analysis showed that habitual consumption of sugar-sweetened beverages was associated with a higher incidence of type 2 diabetes independently of adiposity by 13% (95% confidence intervals, 6 to 21%) [9]. The current consumption of sugar-sweetened beverages was estimated to cause approximately 11% and 5.8% of absolute type 2 diabetes event rates over 10 years in the USA and UK, respectively.

In Japan, the higher consumption frequency of sugar-sweetened beverages in infancy was associated with poor quality of overall dietary intake in 1.5–2.5 years later [10], although the proportional mortality due to sugar-sweetened beverages in the elderly Japanese (> 65 years old) was as low as < 1% compared with Western countries [11]. Approximately 12% of infants consumed sugar-sweetened beverages every day, who had a higher consumption of confectionaries and lower intake of fruits and vegetables in a couple of years later [10]. Soft drink intake was associated with a higher risk of ischemic stroke for women [12]; the multivariable hazard rate for cerebral infarction was 1.83 (95% confidence intervals, 1.22–2.75) among women taking soft drinks almost every day compared to never or rarely. Also, soft drink but not pure fruit/vegetable juice consumption was associated with increased risk of type 2 diabetes in Japanese women [13]. Eshak et al. discussed no consensus about the reason why an adverse effect of soft drink in women was stronger than that in men. Health disorders due to sugar-sweetened beverages will become more problematic in Japan shortly.

According to the 2018 ESC/ESH Guidelines for the management of arterial hypertension and the 2016 European Guidelines on cardiovascular disease prevention in clinical practice, the regular consumption of sugar-sweetened beverages is associated with overweight, metabolic syndrome, type 2 diabetes, and higher cardiovascular disease risk [14, 15]. These guidelines state that the consumption of sugar-sweetened beverages cannot be recommended. The World Health Organization (WHO) guideline highly recommends reducing the intake of sugars to less than 10% of the total energy intake, and this includes added sugars as well as the sugars present in fruits and fruit juices [16].

Several healthy diets that limit an individual’s intake of sweets and sugar-containing beverages are well documented to be effective. The Dietary Approaches to Stop Hypertension (DASH) diet, which is rich in vegetables, fruits, and low-fat dairy products, recommends reducing the consumption of sweets and sugar-containing beverages [17]. The traditional Mediterranean diet, which is characterized by high intakes of olive oil, fruit, nuts, vegetables, cereals, and fish, includes a low consumption of sweets [18]. Both guidelines and intervention studies of healthy diets thus recommend that restricting sugar-containing beverages is essential for maintaining health.

A systematic analysis of the Global Burden of Disease Study 2016 evaluated how much of the disability burden of dementia is affected by sugar-sweetened beverages. The study’s results demonstrated that overall, 28.8 million disability-adjusted life years (DALYs) were attributed to dementia and that 6.4 million DALYs of these (22.3% of the DALYs due to dementia) could be attributed to the modifiable risk factors including a high intake of sugar-sweetened beverages (0.07%) [19]. In that study, a diet high in sugar-sweetened beverages was suggested to be a risk factor for dementia. The effect of a high intake of sugar-sweetened beverages was thought to be mediated with dementia through the BMI, but sugar-sweetened beverages as such explained only a small fraction of the dementia burden that is attributed to risk factors.

Interventions are thus necessary to combat the adverse effects of the consumption of high-sugar and sugar-sweetened beverages on both an individual’s health and a national and global scale, but it is difficult for many people of all ages to reduce (let alone eliminate) their intake of sugar-sweetened beverages. The changes in the use of tobacco products (mainly cigarettes) provide an example of ways to encourage people to stop a bad habit; many government-based public health initiatives have helped create a social environment in which smoking is prohibited or restricted [20]. Regarding beverages, a systematic review published in 2018 showed that the taxation of sweetened beverages significantly influences planned purchases and increases the probability of the purchase of healthy beverages [21]. The review evaluated the impact of a tax on sugar-sweetened beverages in observational studies, and the results indicated that purchases and sales of sugar-sweetened beverages decreased significantly with taxation amounts of 8% (Berkeley, CA) and 10% (Mexico). Sugar-sweetened beverage taxes have the potential to reduce a population’s calorie and sugar intake from a public health and hygiene point of view.

Latin American countries have adopted comprehensive regulations targeting sugar-sweetened beverages for public health reasons [22]. Their five-category framework regulations are as follows: (1) restrictions to the availability of sugar-sweetened beverages in schools, (2) taxes and other economic incentives to discourage consumption, (3) restrictions on advertising and marketing, (4) regulations on government procurement and subsidies, and (5) product labeling rules. In the USA, beverage companies have reduced the sale of sugar-sweetened beverages in schools, limited the television advertising of sugar-sweetened beverages to children, and increased the availability of smaller portion-size options [23].

Although Western countries are working on the social regulation of sugar-sweetened beverages, there has not been any positive regulation of sugar-sweetened beverages in Japan. Social regulations regarding sugar-sweetened beverages are necessary to “put the brakes on” the increases in diabetes morbidity and dementia that often accompanies diabetes.

## Abbreviations

DALYs: Disability-adjusted life years
DASH: Dietary Approaches to Stop Hypertension
ESC: European Society of Cardiology
ESH: European Society of Hypertension
SE: Standard error
UK: United Kingdom
US: United States
WHO: World Health Organization
